# Activation of sperm Toll-like receptor 2 induces hyperactivation to enhance the penetration to mucus and uterine glands: a trigger for the uterine inflammatory cascade in cattle

**DOI:** 10.3389/fimmu.2023.1319572

**Published:** 2023-12-19

**Authors:** Ihshan Akthar, Yejin Kim, Takashi Umehara, Chihiro Kanno, Motoki Sasaki, Mohamed Ali Marey, Mohamed Samy Yousef, Shingo Haneda, Masayuki Shimada, Akio Miyamoto

**Affiliations:** ^1^ Global Agromedicine Research Center (GAMRC), Obihiro University of Agriculture and Veterinary Medicine, Obihiro, Japan; ^2^ Graduate School of Integrated Sciences for Life, Hiroshima University, Higashi-Hiroshima, Japan; ^3^ School of Veterinary Medicine, Kitasato University, Towada, Japan; ^4^ Department of Basic Veterinary Medicine, Obihiro University of Agriculture and Veterinary Medicine, Obihiro, Japan; ^5^ Department of Theriogenology, Faculty of Veterinary Medicine, Damanhur University, Behera, Egypt; ^6^ Department of Theriogenology, Faculty of Veterinary Medicine, Assiut University, Assiut, Egypt; ^7^ Department of Clinical Veterinary Medicine, Obihiro University of Agriculture and Veterinary Medicine, Obihiro, Japan

**Keywords:** cattle, hyperactivation, immune response, mucus, sperm, Toll-like receptor 2, uterine gland

## Abstract

It is known that sperm and seminal plasma (SP) affect uterine immunity. In cattle, artificial insemination enables breeding by depositing frozen and largely diluted sperm with a negligible amount of SP into the uterus. Thus, the present study focused on the impact of frozen-thawed sperm on bovine uterine immunity. We have previously shown that in the bovine uterus, sperm swim smoothly over the luminal epithelium and some sperm interact with uterine glands to induce a weak inflammatory response mainly via the endometrial Toll-like receptor 2 (TLR2) signaling. However, the process by which sperm is encountered in the uterine glands is not completely clear. The present study intended to evaluate the role of sperm-TLR2 in sperm-uterine mucus penetration for reaching the glandular epithelium to induce the uterine immune response. To activate and block sperm-TLR2, they were treated with TLR2 agonist and antagonist, respectively. TLR2 activation enhanced sperm hyperactivation and improved its capacity to penetrate the artificial viscoelastic fluid and estrous-uterine-mucus. In contrast, TLR2-blocked sperm showed completely opposite effects. It is noteworthy, that the TLR2-activated sperm that penetrated the uterine mucus exhibited increased motile activity with hyperactivation. In the sperm-endometrial *ex-vivo* model, a greater amount of TLR2-activated sperm entered the uterine glands with an immune response, which was seen as the upregulation of mRNA expression for *TNFA*, *IL1B*, *IL8*, *PGES*, and *TLR2* similar to those in control sperm. On the other hand, a lesser amount of TLR2-blocked sperm entered the uterine glands and weakened the sperm-induced increase only in *PGES*, suggesting that penetration of a certain number of sperm in the uterine gland is necessary enough to trigger the inflammatory response. Altogether, the present findings indicate that activation of sperm-TLR2 promotes their hyperactivation and mucus penetration with greater motility, allowing them to enter into the uterine glands more. This further suggests that the hyperactivated sperm contributes to triggering the pro-inflammatory cascade partly via TLR2 in the uterus.

## 1 Introduction

In mammals, following natural mating or artificial insemination (AI), semen, which consists of sperm and seminal plasma (SP), enters the female reproductive tract (FRT) (1). During natural mating, in humans and cattle, semen is deposited in the cranial vagina, and from there, sperm migrate to enter the uterine lumen by leaving most of the SP behind in the vagina (1, 2). In pigs and horses, semen is directly deposited into the uterine body (1, 2). In mice, most of the semen is rapidly transported into the uterine cavity, and some remain in the vagina where it coagulates to form a copulatory plug (2). Sperm and SP are known to modulate the immune response of the FRT (3–5).

AI is the most common technique used for breeding cattle worldwide. During AI, sperm are directly deposited into the uterus, with a very low amount of SP, since they are considerably diluted during semen extension with the extender (6). Nevertheless, there are SP components that bind to the surface of sperm and are retained when sperm pass through the uterus (2, 7). Even though millions of sperm enter the uterus, only a few thousand sperm reach the oviduct (1). Our recent research has revealed that following AI, sperm are quickly transported from the site of semen deposition in the uterine body. Within an hour of AI, a large number of sperm pass through the uterine horn, while a massive number of sperm are excluded backward into the vagina. After 6 hours, only a few sperm are found remaining in the uterine lumen (8). Such rapid transport of sperm is independent of the active sperm motility and is regulated by the uterine smooth muscle contractions (9–12). During the sperm passage through the uterus, sperm encounter the uterus’s unique anatomical features such as the microarchitecture and mucus lining the endometrium (13).

The uterus not only facilitates the passage of sperm to the oviduct but also assists in eliminating the huge number of excess and dead sperm through its innate immunity (3). Of note, we have shown that in cattle, sperm interact with glandular epithelium to trigger an acute and transient inflammatory response against sperm (13, 14). Importantly, Toll-like receptor 2 (TLR2) (3, 13–16) in cattle and TLR4 in mice (17) have been recognized as the main mediators of uterine immune responses toward sperm.

TLRs are transmembrane proteins and an essential element of the innate immune system. They play a crucial role in pathogen-specific recognition (18). Even though TLRs are predominantly expressed in immune cells (19), several TLRs are generally present in cells of various tissues including in the male (20) and female reproductive tract (21). In cattle, endometrium expresses TLRs 1 to 10; specifically, TLRs 1 to 7 and 9 are expressed in the endometrial epithelial cells, while TLRs 1 to 4, 6, 7, 9, and 10 are expressed by stromal cells (22). TLR2 is localized in the surface and glandular epithelia of the endometrium of cows (15). In general, TLR2 recognizes various infectious pathogens and their products such as lipoproteins and peptidoglycans (PGN) of Gram-positive bacteria (18). Notably, TLR2 regulates physiological inflammation during fertilization when sperm interact with cumulus-oocyte complexes (23).

TLR2 is expressed also in the sperm of several species such as the Chinese soft-shelled turtle (24), mouse (25), rat (26), and human (27), but the role of TLR2 in sperm is almost unknown. It was reported that the presence of TLR2 in sperm is critical for long-term sperm survival (24) and protection against pathogens (27). It has been shown that sperm-TLR2 activation mediates the motility of sperm in humans and mice (27).

Recently, we investigated the interactions of bovine sperm with the endometrium using the *ex-vivo* sperm-endometrial model. Our results revealed that a few numbers of cultured sperm use their motility to swim smoothly over the luminal epithelium and invade the uterine glands to trigger the inflammatory response (13). This raises the question of which system regulates such particular features of the bovine sperm action to enhance their interaction with the uterine glands. Importantly, we have recently shown that during *in-vitro* fertilization (IVF), sperm-TLR2 activation stimulates sperm penetration to oocytes (28). This led us to hypothesize that sperm may acquire a particular motile pattern that is potentially induced by sperm-TLR2 activation, thereby efficiently penetrating the mucus to reach the glandular epithelium and induce uterine inflammatory responses.

To test the above hypothesis, at first, we confirmed the existence of TLR2 on the bull sperm, and then onwards we examined its impact on sperm motile pattern and subsequent mucus penetration since sperm needs to penetrate the viscoelastic mucus on the endometrial surface before interacting with the uterine glandular epithelium. Then, the impact of TLR2 activation/blockage on the penetration of sperm into the uterine gland and subsequent induction of uterine inflammatory response was examined.

## 2 Materials and methods

### 2.1 Experimental model

**Figure 1:**
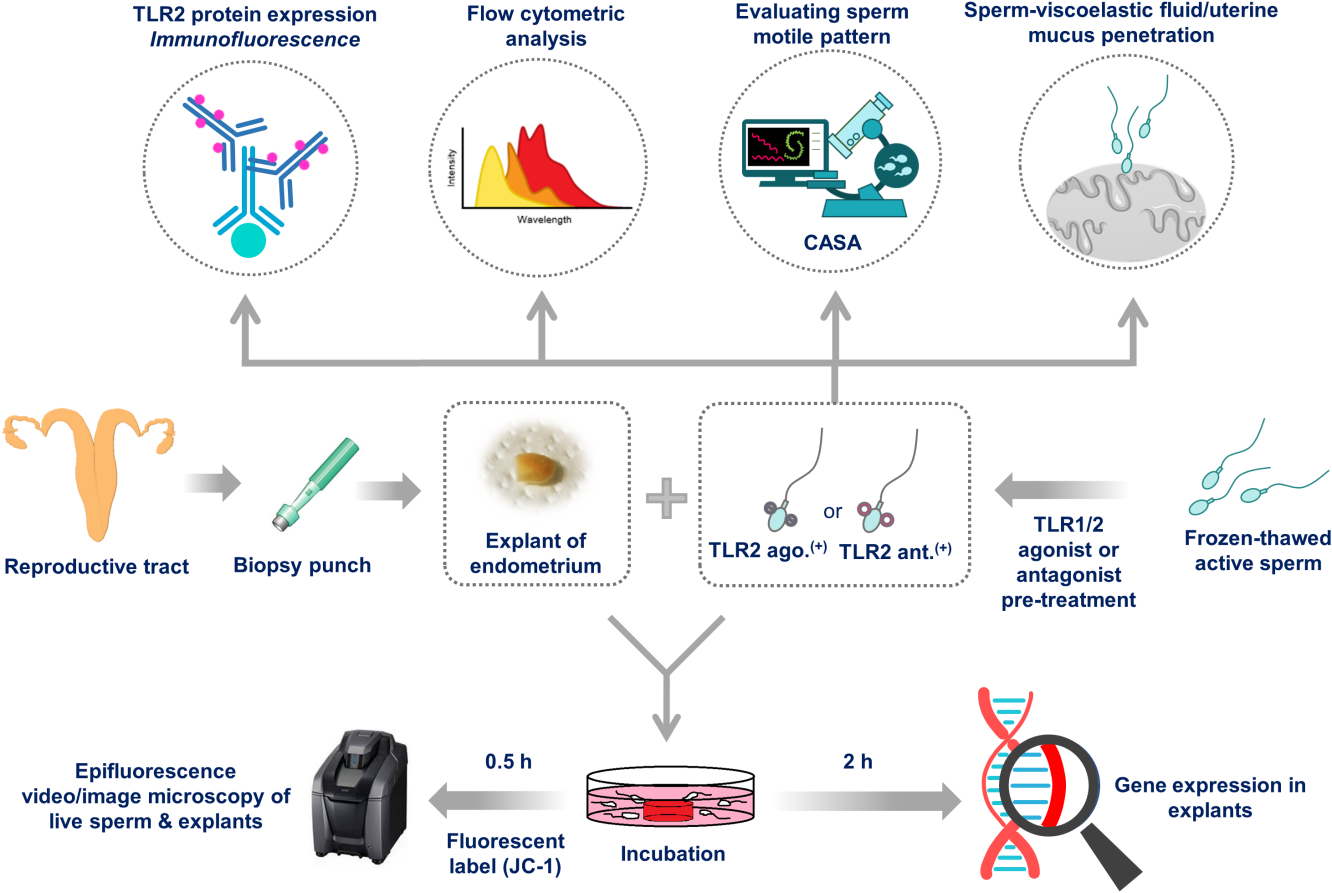
Schematic illustration of the experimental model. Initially, the localization of TLR2 in the frozen-thawed active bull sperm was analyzed using immunofluorescence staining. Frozen-thawed active (swim-up) bull sperm were treated with TLR1/2 agonist (to activate the TLR2) or antagonist (to block the TLR2). Sperm motion parameters and organelle functions were evaluated by CASA and flow cytometry analysis, respectively, to evaluate the effects of TLR2 activation and blockage on sperm. Since the sperm needs to penetrate the viscoelastic uterine mucus before entering into the uterine glands, at first, the specific sperm motile pattern (hyperactivated sperm) responsible for penetrating the mucus was calculated. Then, to observe the effect of activation of sperm-TLR2 on mucus penetration, percentages of sperm that penetrated the viscoelastic fluid (0.7% long-chain polyacrylamide, LC-PAM) and estrous-uterine-mucus were evaluated. Moreover, sperm motion patterns of uterine mucus-penetrated sperm were evaluated. Further, bovine endometrial explants were incubated with JC1-labeled sperm, and sperm-uterine gland interactions were observed using fluorescence microscopy. To evaluate the impact of sperm-TLR2 on uterine immune responses, TLR2-activated and -blocked sperm were incubated with endometrial explants, and mRNA expression of pro-inflammatory cytokines, *PGES*, and *TLR2* was evaluated.

The localization of TLR2 on sperm was evaluated using immunofluorescence staining. The impact of TLR2 activation and blockage on sperm were analyzed via flow cytometry and computer-assisted sperm analysis (CASA). The impact of sperm-TLR2 on mucus penetration as well as sperm penetration of the viscoelastic fluid and estrous-uterine-mucus was evaluated by the CASA outcomes. Then, we evaluated the role of sperm-TLR2 in sperm-uterine immune interactions, using the *ex-vivo* co-incubation model of sperm and endometrial tissue. The detailed experimental model is shown in **Figure 1**.

### 2.2 Reagents and media

Chemicals were procured from Wako Pure Chemical Industries (Japan) except otherwise stated. The TALP (modified Tyrode balanced salt solution) (29) was used to wash and dilute sperm, as well as to incubate sperm with endometrial explants (13). The viscoelastic model mucus was prepared by adding Long-chain polyacrylamide, LC-PAM (5-6 MDa, Sigma-Aldrich, USA) into the TALP. The TALP and the model mucus were balanced in a 38.5°C incubator with 5% CO_2_.

### 2.3 Sperm sample preparation

The swim-up technique was used to separate the active sperm from the frozen-thawed semen (30). Briefly, frozen semen packed in 0.5 ml straws derived from three fertile Holstein bulls were used. The semen was collected and processed by the Genetics Hokkaido Association (Hokkaido, Japan) under controlled hygienic measures. The frozen-thawed semen was confirmed to be free from bacterial contamination. At first, sperm (0.25 ml) was layered under TALP (1 ml). After 1 h of incubation, the top sperm layers (0.5 ml) were collected, pooled, and washed at 200g for 5 min. To activate the TLR2 (15, 27, 28) in sperm, TLR1/2 agonist, Pam3Cys-Ser-(Lys)4 (Pam, ab142085, Abcam) (a synthetic analog of the triacylated N-terminal part of gram-positive bacterial peptidoglycan) was used. Initially, 100 µg/ml of stock agonist solution in 50% ethanol was prepared. To obtain TLR2-activated sperm (TLR2 ago.^(+)^), 100 ng/ml of agonist was incorporated into the sperm and incubated further for 2 h in a 38.5˚C incubator. Then, two times centrifugation at 200g for 10 min in 10 ml TALP was conducted to remove the agonist. Then, the sperm was suspended in TALP. TLR2 non-activated sperm (TLR2 ago (–).) were prepared in the same way (with the appropriate vehicle; 50% ethanol for TLR1/2 agonist) except without agonist. To block the TLR2 (15, 28) in sperm, TLR1/2 antagonist, a synthetic TLR2 blocker (CU-CPT22; Merck, Darmstadt, Germany) was used (31). Initially, 100 mM of stock antagonist solution in DMSO was prepared. To obtain TLR2-blocked sperm (TLR2 ant.^(+)^), 100 µM of the antagonist was incorporated into the sperm and incubated further for 30 min in a 38.5˚C incubator. Then, two times centrifugation at 200g for 10 min in 10 ml TALP was conducted to remove the antagonist. The resultant sperm were re-suspended in TALP. TLR2 non-blocked sperm (TLR2 ant (–).) were prepared in the same way (with the appropriate vehicle; DMSO for TLR1/2 antagonist) except without antagonists. The concentrations of the agonist and antagonist were chosen based on an earlier study (28). Sperm cell concentration was obtained via a hemocytometer (C-chip, NanoEnTek, Korea). To evaluate the sperm plasma membrane and acrosomal integrity (PMAI), high mitochondrial membrane potential (HMMP), plasma membrane stability, and sperm motion parameters, sperm were incubated for 0, 0.5, and 2 h in a 38.5˚C incubator.

### 2.4 Immunofluorescence analysis

Immunofluorescence was conducted to detect the TLR2 protein localization in bull sperm based on a previously described protocol (24) with modifications. The sperm (5x10^6^ cells/ml) were spread on slides (S8226, Matsunami Glass Int., Osaka, Japan), air-dried at room temperature (RT), and fixed in 10% formalin in phosphate buffer (PB) at RT. After 10 min, the samples were immersed in absolute methanol for 5 min at -20˚C. Then, the samples were blocked with 5% BSA for 30 min at RT. Afterward, the slides were incubated overnight (4˚C in a humidified chamber) with the primary antibody (1:50, 10 µg/ml, rabbit polyclonal anti-TLR-2orb11487, Biorbyt, Cambridge, UK). Then, the sections were labeled with the secondary antibody (1:200, 10 µg/ml, goat anti-rabbit IgG labeled with Alexa Fluor 546, Invitrogen, Thermo Fisher Scientific, USA) for 1 h at RT. The samples were labeled with DAPI (1:50, 340-07971, Dojindo Laboratories, Japan) for 30 min. Slides were washed and mounted using VECTASHIELD mounting medium (H-1000; Vector Laboratories, Burlingame, CA 94010, USA). The Rabbit IgG isotype (1:500, 10 µg/ml, Invitrogen, Thermo Fisher Scientific) was applied as the IgG negative control. The sections were observed via fluorescence microscope (Keyence, BZ-X800, Osaka, Japan) through the BZ-X TexasRed (red wavelength) and BZ-X DAPI (blue wavelength) filters. Exposure time was maintained uniformly for all the sections.

### 2.5 Evaluation of plasma membrane and acrosomal integrity, high mitochondrial membrane potential, and plasma membrane stability of sperm by flow cytometry

The sperm PMAI and HMMP were assessed based on previous reports with minor modifications (32–34). Briefly, each treatment aliquot was labeled with Propidium Iodide (PI, 5 µg/ml, Sigma Aldrich) and Fluorescein isothiocyanate-conjugated peanut-agglutinin (FITC-PNA, 25 µg/ml, Vector Laboratories). PI and FITC-PNA negative cells were measured as sperm with intact plasma membrane and acrosome (PMAI). For evaluation of HMMP, each treatment aliquot was stained with 5,5’,6,6’-tetrachloro-1,1’3,3’-tetraethylbenzimidazolyl-carbocyanine iodide (JC1) fluorescent probe (5 µM, AdipoGen). The sperm emitting orange fluorescence were considered sperm with HMMP. The sperm plasma membrane stability of viable sperm was analyzed according to an earlier report (35). Each treatment aliquot was stained with cell viability indicator Yo-Pro 1 (50 nM, Y3603, Invitrogen) and plasma membrane destabilization indicator Merocyanine 540 (M540, 2.7 µM, 323756, Sigma Aldrich). Yo-Pro 1 and M540 negative cells were considered viable sperm with a stable plasma membrane. Percentages of sperm with stable plasma membranes were calculated out of total viable sperm. The labeled suspensions were incubated at 38.5˚C for 10 min under dark conditions. Sperm suspensions were analyzed using a spectral cell analyzer (Sony SA3800, Sony Imaging Products & Solutions., Tokyo, Japan). Each fluorochrome was excited by 488 nm laser, and emissions were detected on PMT channel 5-10 (515-546 nm, FITC), 18-19 (598-617 nm, PI), 5-8 (515-534 nm, JC1 Green), 15-17 (573-598 nm, JC1 Orange), 2 (503-507 nm, Yo-Pro 1), and 16 (581-589 nm, M540). The flow cytometer was used at short boost mode and a low flow rate of one. The acquisition was halted after recording 10,000 single sperm events. The representative flow cytometric dot plot diagrams are given in **Supplementary Figure 1**.

### 2.6 Assessment of sperm motion parameters by CASA

CASA was conducted according to an earlier report (36). A measurement of 3 µL (10 x 10^6^ cells/ml) of sperm was added onto a warmed (38.5°C) chamber slide (SC20-01-04-B, Leja, GN Nieuw-Vennep, Netherlands). More than 200 sperm tracks were tracked via the CASA analyzer (SMAS, DITECT, Tokyo, Japan) at 150 frames per second (fps), depending on the images gained through a phase-contrast microscope (ECLIPSE Ci-L, Nikon, Tokyo, Japan) in each replicate. The experiment was repeated five times. The percentage of total, progressive, hyperactivated motile sperm, straight-line velocity (VSL), curvilinear velocity (VCL), average path velocity (VAP), linearity (LIN), straightness (STR), beat cross frequency (BCF), and amplitude of lateral head (ALH) were measured.

### 2.7 Evaluation of hyperactivated sperm

It was reported that, in humans, sperm that could penetrate periovulatory cervical mucus had an increased velocity and side-to-side head movement, with a similar group of kinetic properties with VAP ≥ 25.0 µm/s and ALH ≥ 7.5 µm (37, 38). Moreover, sperm showing VCL ≥ 150 µm/s, LIN ≤ 50%, and ALH ≥ 7.0 µm were classified as hyperactivated sperm (39). In cattle, sperm with increased mean VCL and ALH and decreased LIN were characterized as hyperactivated sperm (40), and these sperm are known to advance through the viscoelastic mucus of the female tract and matrix of the cumulus oophorus (41). These reports indicate that hyperactivated sperm can penetrate the mucus efficiently. Therefore, in the present study, the hyperactivated sperm were characterized as sperm that could penetrate the mucus to a greater extent when compared to the non-hyperactivated sperm and computed based on the sperm motion parameters VCL, ALH, and LIN.

At first, threshold values for hyperactivated sperm (41) were evaluated with calcium ionophore (A23187)-induced hyperactivation (42). Briefly, 5 x 10^6^ cells/ml of sperm were treated with A23187 (final concentration 1µM, C7522, Sigma Aldrich) for 30 min, washed at 200g for 10 min, and evaluated via CASA (SMAS, DITECT, Tokyo, Japan) at 150 frames per second (fps). Two observers independently evaluated at least 200 individual sperm tracks. The experiment was repeated three times with different sperm suspensions. Based on the individual sperm trajectories, hyperactivated sperm were judged and the VCL, ALH, and LIN of each hyperactivated sperm were recorded as previously suggested (41). Since all the sperm that were judged as hyperactivated showed VCL ≥ 200 µm/s, ALH ≥ 3 µm, and LIN ≤ 40%, these values were set as the threshold values for hyperactivated bull sperm in our CASA system (SMAS, DITECT, Tokyo, Japan). Then, the percentage of hyperactivated sperm along with other sperm motility parameters were measured in suspensions of interest.

### 2.8 Sperm-viscoelastic fluid or -estrous-uterine-mucus penetration assay

We investigated the influence of sperm-TLR2 on the ability of sperm to penetrate mucus via sperm-model viscoelastic fluid and -estrous-uterine-mucus penetration assays.

We used 0.7% of LC-PAM in the TALP medium as the model viscoelastic mucus. This media mimics the bovine estrous cervical mucus (43, 44). Since the overall mucus in the uterus may contain the majority of the cervical mucus (2) and the endometrial secretion, we used 0.7% LC-PAM solution for comparison with the mucus of the uterus. The percentages of viscoelastic fluid penetrated sperm were assessed by layering sperm over a viscoelastic fluid layer in a 1.5 ml microcentrifuge tube. Phenol red (1 mg/ml) was incorporated into the viscoelastic fluid to differentiate the sperm suspension and viscoelastic fluid layers. A measurement of 1 ml of viscoelastic fluid was placed in a microcentrifuge tube. A measurement of 0.5 ml of 10x10^6^ cells/ml sperm suspension was gently layered over the viscoelastic fluid layer and incubated at 38.5°C incubator for 30 min. After the incubation period, the top 0.5 ml of the sperm suspension layer was gently aspirated. Additionally, 0.1 ml of the top viscoelastic fluid layer was aspirated to avoid the mixing of sperm suspension with the viscoelastic fluid layer. The remaining 0.9 ml of viscoelastic fluid was mixed and sperm concentration in the viscoelastic fluid was evaluated. Based on initial sperm concentration in the sperm suspension and final sperm concentration in the viscoelastic fluid layer, the proportion of viscoelastic fluid-penetrated sperm was calculated.

The estrous-uterine-mucus was obtained from pre-ovulatory stage cows through mechanical pressure. The uterine mucus was centrifuged twice at 7200g for 10min at 4°C and used in the sperm-mucus penetration assay as mentioned above. The 0.5 ml of uterine mucus was placed in a microcentrifuge tube and 0.25 ml of 10x10^6^ cells/ml sperm was gently layered over the uterine mucus layer and incubated at 38.5°C for 30 min. After the incubation period, the top 0.25 ml of the sperm layer was gently aspirated. Additionally, 0.05 ml of the top uterine mucus layer was aspirated to avoid mixing the sperm suspension with the uterine mucus layer. The remaining 0.45 ml of uterine mucus was mixed and sperm concentration and motion behavior (via CASA) in the uterine mucus were evaluated. Based on the initial sperm concentration in the sperm suspension and final sperm concentration in the uterine mucus layer, the proportion of uterine mucus penetrating sperm was calculated. The estrous-uterine-mucus obtained from five different cows was used in the five repeats of this experiment.

### 2.9 Animals and tissue preparation

The healthy bovine female reproductive tracts were obtained from the abattoir (Doto plant Tokachi Factory, Obihiro, Japan). The phase of the tract was identified based on a previous report (45). The ipsilateral ovarian follicle-containing horns from the preovulatory stage (days 19-22) were separated and transported on ice-cold saline. Endometrial explants were extracted and incubated based on an earlier defined protocol (13). Briefly, with the aid of a biopsy punch, explants were extracted from the glandular endometrial regions and pre-incubated in a 38.5˚C incubator for 15 min before starting the co-incubation with sperm.

### 2.10 Determination of sperm numbers within uterine glands

As per the previous description, we evaluated the number of sperm within the uterine glands (13). Briefly, JC1-stained sperm (10^6^ cells/ml) were cultured with endometrial explants. After 30 min, sperm in endometrial explants were viewed using the fluorescence microscope, and sperm numbers within the uterine glands were assessed. Videos and images were taken using the BZ-X TexasRed (red wavelength) and BZ-X GFP (green wavelength) filters. Three glands were assessed in each treatment; the experiment was repeated five times using uteri explants obtained from different cows.

### 2.11 The immune response of sperm in endometrial explants

The immune response of endometrial explants to TLR2-activated or blocked sperm was investigated based on a previously described method (13). Briefly, explants were co-cultured with TLR2-activated or blocked sperm (10^6^ cells/ml) for 2 h then the explants were collected and washed three times in TALP to get rid of sperm and stored in TRIZOL at -80˚C for total RNA extraction. After washing, very few sperm remained within the uterine glands; thus, the explant preparation for analysis of mRNA expression contained very few sperm. Our group previously showed that sperm express a negligible amount of mRNA for our selected genes (30), so the changes in mRNA expression of these genes were only attributed to uterine explants in response to sperm.

### 2.12 RNA extraction, cDNA synthesis, and qPCR

Endometrial explants were homogenized (13) and RNA extraction was conducted based on a previously described protocol (46). Prior to cDNA synthesis, NanoDrop Spectrophotometer (2000c, Thermo Scientific, Waltham, MA, USA) was used to obtain the concentration and purity of RNA. The cDNA was synthesized following a previously described protocol (47).

Quantitative real-time PCR was conducted by a MiniOpticon (Bio-Rad Laboratories, Tokyo, Japan) via SYBR Green PCR Master Mix (Bio-Rad Laboratories, USA). The targeted primer pairs are listed in **Supplementary Table 1**. The amplification program was set up according to a previously described protocol (13). The melting curve was evaluated at the end of the run to observe the specificity of the amplification. A negative control, reaction-containing nuclease-free water or non-reverse transcribed RNA was incubated in each run. The housekeeping gene, *β-actin*, was used as an internal standard for normalization of Ct values. The Delta-Delta comparative threshold method was used to determine the fold changes in relative mRNA expression and these fold changes were used in the statistical analysis (48).

### 2.13 Statistical analyses

GraphPad Prism 5 software was applied for statistical analysis (GraphPad Software, La Jolla, CA, USA). The data in all experiments were normally distributed. The mean differences between the two groups were compared using the student t-test. The mean differences for more than two groups were compared using one-way ANOVA followed by Tukey’s tests. For mRNA expression, the animal was selected as a statistical unit and each experiment was repeated five times using explants from five different uteri (three explants/treatment/experiment). Each treatment had three replicates, and the mean of these replicates of each uterus was considered and these values were applied in the analysis and depicted in the figures. The values are presented as mean ± standard error of the mean (SEM). The significance of the data was determined based on their respective p-values where *P<0.05, **P<0.01, and ***P<0.001. All experiments were repeated five times.

## 3 Results

### 3.1 Immunofluorescence localization of TLR2 in bull sperm

**Figure 2:**
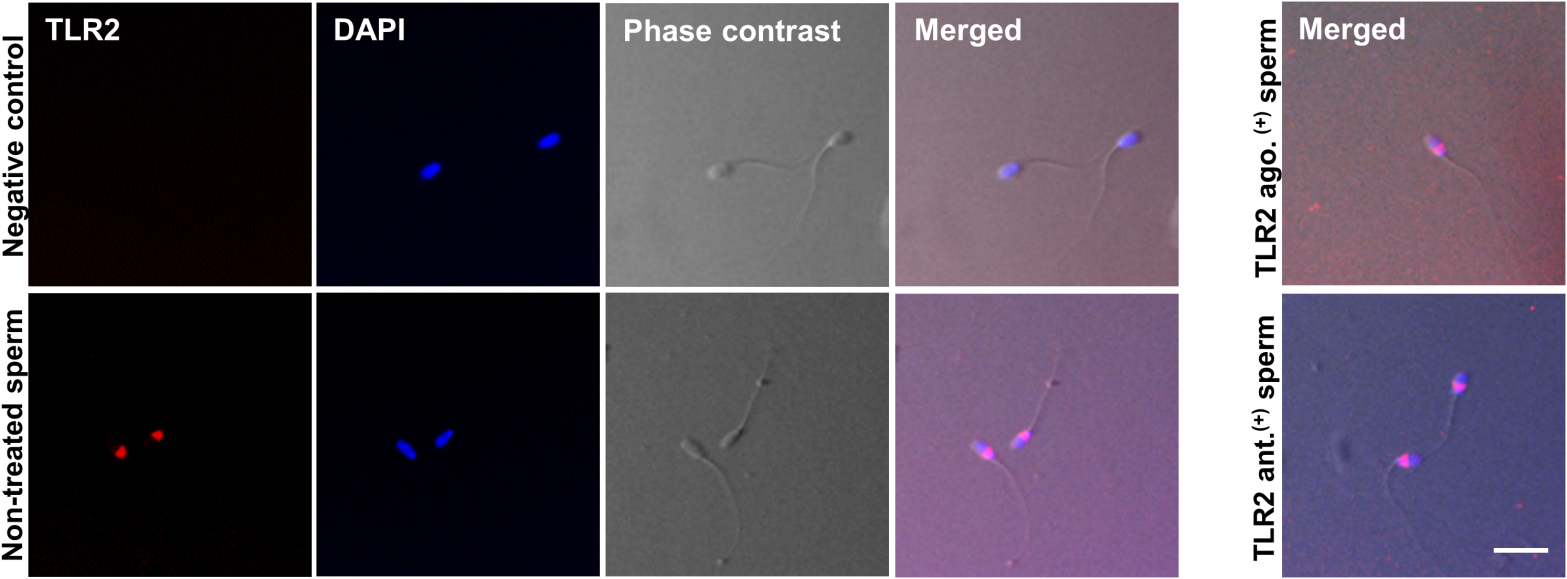
Immunofluorescence localization of TLR2 in bull sperm. The micrographs show the localization of TLR2 in non-treated, TLR2-activated (TLR2 ago.^(+)^), and TLR2-blocked (TLR2 ant. ^(+)^) bull sperm. The localization was detected via immunofluorescence labeling with the Alexa-Fluor-conjugated anti-TLR2 antibody. For negative control, the sections were treated with Rabbit IgG isotype. DAPI stains the nucleus. The phase-contrast micrographs depict the morphology of the bull sperm. TLR2 was expressed in the posterior segment of the non-treated bull sperm head in all stained sperm. TLR2 localization did not change with the TLR2 activation or TLR2 blockage and was expressed in the same location as non-treated sperm. The result is representative of five separate experiments. In each experiment, more than 200 sperm per group were evaluated. Scale bar = 20µM.

Immunofluorescence analyses showed TLR2 protein localization in the bull sperm head’s posterior segment in all stained cells. The TLR2 activation or blockage did not change the TLR2 expression in the sperm head. The IgG controls did not show any fluorescent signal (**Figure 2**).

### 3.2 TLR2 activation or blockage did not affect the PMAI, HMMP, plasma membrane stability, and mean values of sperm motion parameters

Since sperm PMAI, HMMP, and plasma membrane stability were the important functional prerequisites for sperm action, we aimed to test whether TLR2 agonist (to activate TLR2) or antagonist (to block TLR2) had any negative effects on these sperm parameters via flow cytometric analysis. Moreover, mean values of sperm motion parameters were analyzed via CASA.

According to the flow cytometry analysis and CASA, neither the TLR2 activation nor blockage could affect the PMAI, HMMP, and plasma membrane stability (**Supplementary Tables 2 and 3**), as well as the percentage of total, progressive motile sperm and mean values of sperm motion parameters such as VSL, VCL, VAP, LIN, STR, BCF, and ALH of sperm when compared to non-treated sperm at 0, 0.5, and 2 h (**Supplementary Tables 4 and 5**).

### 3.3 TLR2 activation and blockage differentially modulated the hyperactivated sperm motility

The endometrial surface was coated with the viscoelastic mucus layer, and sperm had to penetrate this mucus before entering the uterine glands. Since sperm with hyperactivated motility were known to penetrate the mucus efficiently, we investigated whether regulation of sperm-TLR2 modulated sperm hyperactivation which was related to mucus penetration.

**Figure 3:**
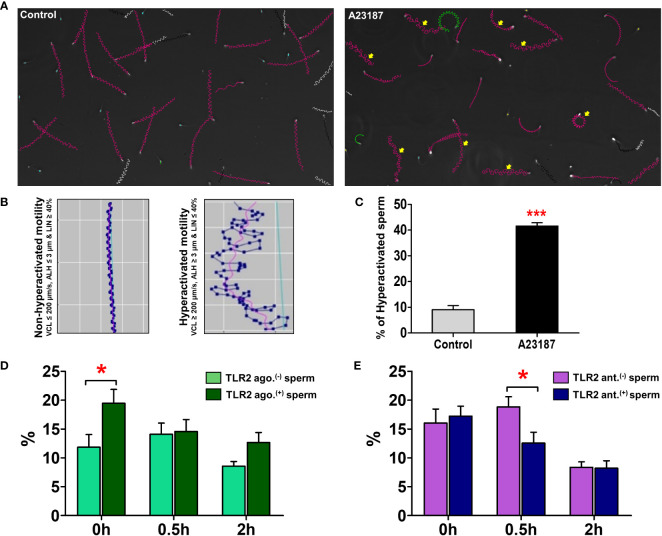
Sperm-TLR2 activation enhanced the hyperactivated sperm motility. **(A)** Sperm trajectory profiles of control and Calcium ionophore (A23187)-treated sperm. The arrows indicate the hyperactivated sperm trajectories. **(B)** Individual sperm trajectories of non-hyperactivated (VCL ≤ 200 µm/s, ALH ≤ 3 µm, and LIN ≥ 40%) and hyperactivated (VCL ≥ 200 µm/s, ALH ≥ 3 µm, and LIN ≤ 40%) sperm. **(C)** The percentage of hyperactivated sperm in control and A23187-treated groups. Data are presented as mean ± SEM of three independent experiments. In each experiment, 200 individual sperm tracks were evaluated. ***p<0.0001 denotes the significant difference. **(D)** The percentage of hyperactivated sperm in TLR2 non-activated (TLR2 ago (–).) and TLR2-activated (TLR2 ago.^(+)^) groups. **(E)** The percentage of hyperactivated sperm in TLR2 non-blocked (TLR2 ant (–).) and TLR2-blocked (TLR2 ant.^(+)^) groups. Data are presented as mean ± SEM of five independent experiments. In each experiment, 200 individual sperm tracks were evaluated. *p<0.05 denotes the significant difference.

Initially, we determined the threshold values for hyperactivated sperm in the CASA system (please refer to the methodology section for more details). To do this, a calcium ionophore (A23187) was utilized to induce hyperactivation. All sperm with hyperactivation showed VCL ≥ 200 µm/s, ALH ≥ 3 µm, and LIN ≤ 40% (**Figures 3A, B**). As a result, these values were defined as the threshold values for hyperactivated bull sperm. The A23187 resulted in an increase in the mean values of VCL and ALH and a decrease in LIN (p<0.05) (**Supplementary Table 6**). The A23187 treatment pointedly enhanced the percentage of hyperactivated sperm compared to the control (p<0.001) (**Figure 3C**).

Analyzing sperm motility revealed that a small portion of sperm (10-15%) acquired hyperactivation without any stimulus (**Figures 3D, E**). Notably, TLR2 activation increased the percentage of hyperactivated sperm at 0h compared to the non-activated sperm (p<0.05). However, activation of TLR2 did not modulate the percentage of hyperactivated sperm at 0.5 and 2h (**Figure 3D**). Meanwhile, TLR2 blockage reduced the percentage of hyperactivated sperm at 0.5h (p<0.05). The TLR2 blockage did not modulate the percentage of hyperactivated sperm at 0 and 2h (**Figure 3E**).

### 3.4 TLR2 activation and blockage impacted the sperm viscoelastic fluid penetration

**Figure 4:**
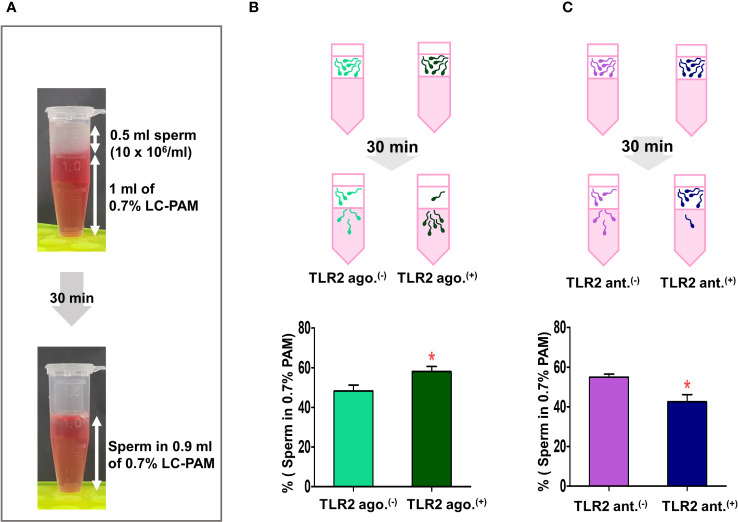
Sperm-TLR2 activation induced the sperm penetration of the viscoelastic fluid. **(A)** Brief methodology showing sperm penetrating the viscoelastic fluid (0.7% of long-chain polyacrylamide, LC-PAM). The number of sperm that penetrated the viscoelastic fluid was evaluated after 30 min following incubation of 10x10^6^ cells/mL (0.5 ml) of sperm layering over the 1 ml of LC-PAM. **(B)** The percentage of penetrated TLR2 non-activated (TLR2 ago (–).) and TLR2-activated (TLR2 ago.^(+)^) sperm into the 0.9 ml LC-PAM fraction. **(C)** The percentage of penetrated TLR2 non-blocked (TLR2 ant (–).) and TLR2-blocked (TLR2 ant.^(+)^) sperm in the 0.9 ml LC-PAM fraction. Data are presented as mean ± SEM of five independent experiments. *p<0.05 denotes the significant difference.

Since the endometrial surface was coated with a viscoelastic mucus layer, we tested whether sperm-TLR2 regulated sperm penetration to mucus via sperm-model viscoelastic fluid penetration assay (**Figure 4A**).

In this assay, a significantly higher percentage of TLR2-activated sperm penetrated the viscoelastic fluid when compared to the non-activated sperm (p<0.05, ~ 20% increase *vs.* TLR2 non-activated sperm) (**Figure 4B**). Meanwhile, a lesser percentage of TLR2-blocked sperm penetrated the viscoelastic fluid when compared to the non-blocked sperm (p<0.05, ~ 22% reduction *vs.* TLR2 non-blocked sperm) (**Figure 4C**).

### 3.5 TLR2 activation enhanced the sperm penetration and motility in estrous-uterine-mucus

**Figure 5:**
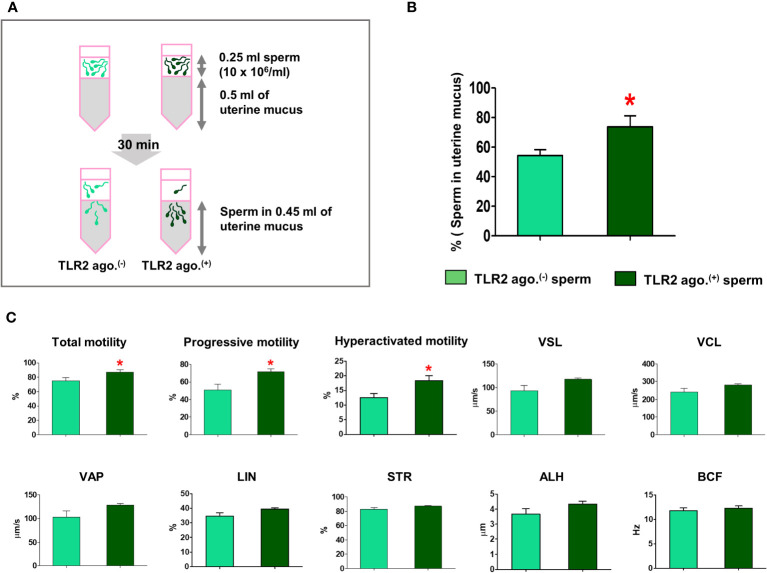
Sperm-TLR2 activation enhanced sperm penetration and motile activity in the estrous-uterine-mucus. **(A)** Brief methodology showing sperm penetrating the estrous-uterine-mucus. The number of sperm and sperm motion parameters that penetrated the estrous-uterine-mucus was evaluated after 30 min following incubation of 10x10^6^ cells/mL (0.25 ml) of sperm layering over the 0.5 ml of uterine mucus. **(B)** The percentage of penetrated TLR2 non-activated (TLR2 ago (–).) and TLR2-activated (TLR2 ago.^(+)^) sperm into the 0.45 ml uterine mucus fraction. Data are presented as mean ± SEM of five independent experiments. *p<0.05 denotes the significant difference. **(C)** The motion parameters of estrous-uterine-mucus penetrated TLR2 ago (–). and TLR2 ago.^(+)^ sperm analyzed via CASA. Data are presented as mean ± SEM of five independent experiments. In each experiment, 200 individual sperm tracks were evaluated. *p<0.05 denotes the significant difference.

Since the sperm-TLR2 regulated the sperm penetration to model viscoelastic fluid, we, therefore, tested the sperm-TLR2 activation on sperm penetration to estrous-uterine-mucus (**Figure 5A**) and subsequent sperm motility behavior in this mucus. A significantly higher percentage of TLR2-activated sperm penetrated the estrous-uterine-mucus when compared to the non-activated sperm (p<0.05, ~ 36% increase *vs.* TLR2 non-activated sperm) (**Figure 5B**).

Meanwhile, the percentage of total, progressive, and hyperactivated motility of TLR2-activated sperm that penetrated the estrous-uterine-mucus was significantly increased compared to the non-activated sperm (p<0.05). However, the mean values of other motion parameters such as VSL, VCL, VAP, LIN, STR, BCF, and ALH of TLR2-activated sperm were not affected in estrous-uterine-mucus (**Figure 5C**).

### 3.6 TLR2 activation and blockage modulated the presence of sperm in uterine glands

**Figure 6:**
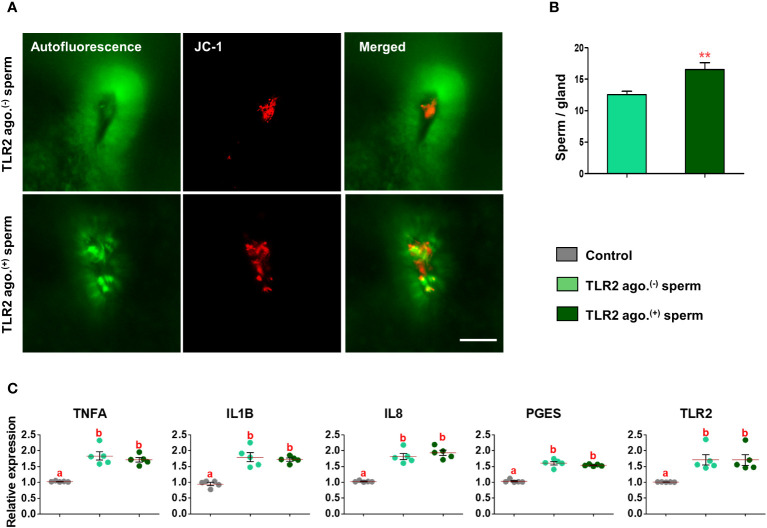
Sperm-TLR2 activation increased sperm numbers in glands and modulated the subsequent sperm-induced mRNA expression in explants. **(A)** JC1 stained midpiece mitochondria of TLR2 non-activated (TLR2 ago.^(-)^) and TLR2-activated (TLR2 ago.^(+)^) sperm within the uterine glands after 30 min incubation with endometrial explants. Bar = 50µM. **(B)** The number of sperm/glands in explants incubated with TLR2 ago.^(-)^ and TLR2 ago.^(+)^ sperm. Data are presented as mean ± SEM of five independent experiments. **p<0.01 denotes the significant difference. **(C)** Relative mRNA expression of pro-inflammatory cytokines, *IL8*, *PGES*, and *TLR2* in endometrial explants incubated with 10^6^ cells/mL TLR2 ago.^(-)^, TLR2 ago.^(+)^, and without sperm (control) for 2 (h) Data are presented as mean ± SEM of five independent experiments. Three uterine explants for each treatment were used from an individual cow in each experiment. Different letters denote a significant difference (p<0.05).

**Figure 7:**
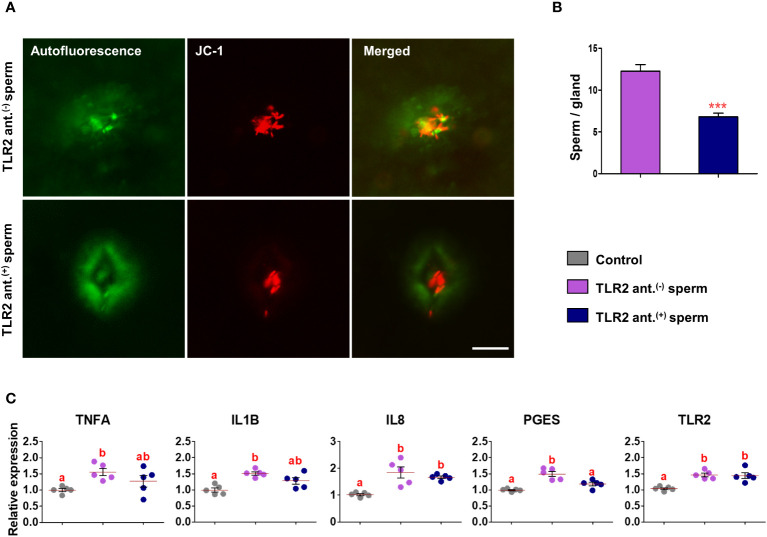
Sperm-TLR2 blockage reduced sperm numbers in glands and modulated the subsequent sperm-induced mRNA expression in explants. **(A)** JC1 stained midpiece mitochondria of TLR2 non-blocked (TLR2 ant.^(-)^) and TLR2-blocked (TLR2 ant.^(+)^) sperm within the uterine glands after 30 min incubation with endometrial explants. Bar = 50µM. **(B)** The number of sperm/glands in explants incubated with TLR2 ant.^(-)^ and TLR2 ant.^(+)^ sperm. Data are presented as mean ± SEM of five independent experiments. ***p<0.001 denotes the significant difference. **(C)** Relative mRNA expression of pro-inflammatory cytokines, *IL8*, *PGES*, and *TLR2* in endometrial explants incubated with 10^6^ cells/mL of TLR2 ant.^(-)^, TLR2 ant.^(+)^, and without sperm (control) for 2 (h) Data are presented as mean ± SEM of five independent experiments. Three uterine explants for each treatment were used from an individual cow in each experiment. Different letters denote a significant difference (p<0.05).

Fluorescence microscopy revealed that 30 min after the co-incubation of TLR2-activated or blocked sperm with endometrial explants, the sperm entered the uterine glands (**Figures 6A** and **7A**). A higher number of TLR2-activated sperm entered and remained within the uterine glands when compared to TLR2 non-activated sperm (p<0.01, ~ 32% increase *vs.* TLR2 non-activated sperm) (**Figures 6A, B**). A lesser number of TLR2-blocked sperm remained in the uterine glands when compared to TLR2 non-blocked sperm (p<0.001, ~ 44% reduction *vs.* TLR2 non-blocked sperm) (**Figures 7A, B**).

### 3.7 TLR2 activated and blocked sperm differently modulated the immune transcription in endometrial explants

Incubation of TLR2 non-activated or non-blocked sperm with endometrial explants upregulated the mRNA expression of tumor necrosis factor-alpha (*TNFA*), interleukin 1-beta (*IL1B*), interleukin 8 (*IL8*), prostaglandin E synthase (*PGES*), and *TLR2* compared to the control (p<0.05) (**Figures 6C** and **7C**). Incubation of TLR2-activated sperm with endometrial explants did not further upregulate the mRNA expression of observed genes (**Figure 6C**), whereas the exposure to TLR2-blocked sperm inhibited the sperm-induced increase in *PGES* mRNA expression (p<0.05) (**Figure 7C**). Moreover, TLR2-blocked sperm in comparison to the control did not upregulate the *TNFA* and *IL1B* mRNA expressions, whereas they upregulated the *IL8* and *TLR2* mRNA expressions (p<0.05) (**Figure 7C**).

## 4 Discussion

**Figure 8:**
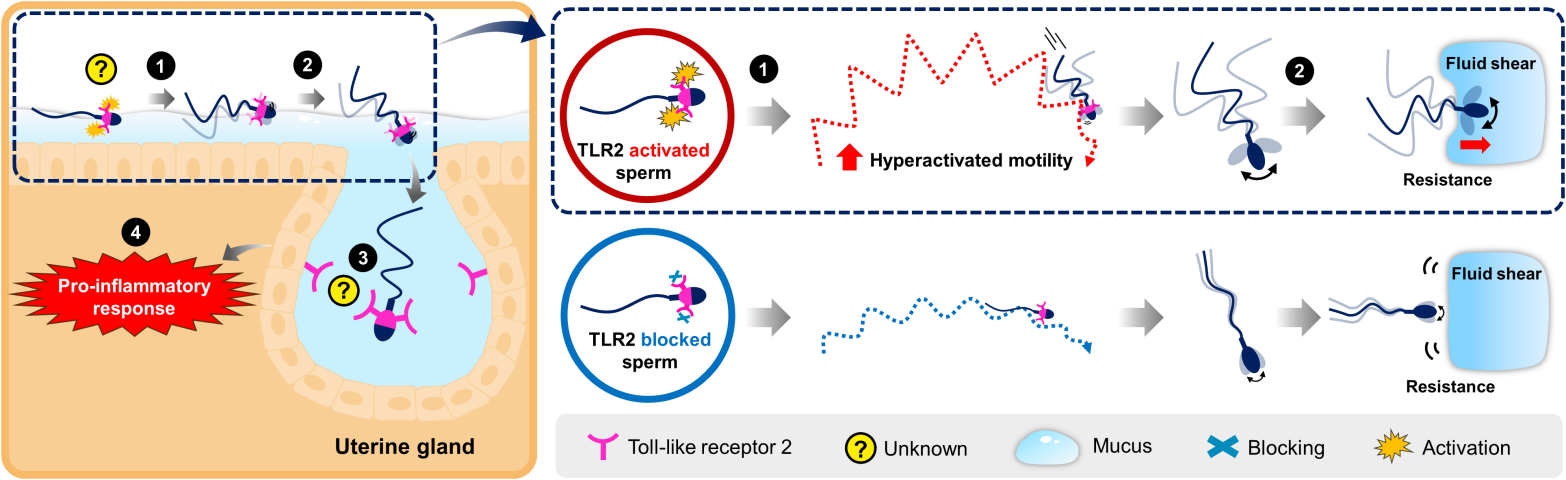
Schematic illustration for the working hypothesis of the interaction between bovine sperm and uterine gland to induce the inflammatory response. The Toll-like receptor 2 (TLR2) is localized in the posterior segment of the bull sperm head. In the uterus, sperm glide over the mucus layer on the surface epithelium while migrating toward the oviduct. (1) Activation of sperm-TLR2, possibly by the endogenous TLR2 ligands regulates the sperm motile pattern by increasing hyperactivated motility. (2) Presumably, these hyperactivated sperm overcome the greater resistance and fluid shear to penetrate through the viscoelastic estrous-uterine-mucus and enter into the uterine glands. (3) Subsequently, this sperm interacts with the uterine gland (4) to trigger the inflammatory response towards sperm. However, the detailed molecular binding mechanism of sperm-TLR2 in the direct interaction with the uterine gland epithelium is unknown and yet to be investigated.

In the uterus, sperm are rapidly transported in the uterine cavity through the contraction of uterine smooth muscles. For the first time, the present investigation demonstrates that sperm undergo hyperactivation via TLR2 and utilize the activated motility to penetrate the lining uterine mucus and invade the uterine glands to induce transient uterine inflammatory responses in cattle. The findings suggest that this sperm-TLR2-mediated hyperactivation may occur in the uterus after insemination. The working hypothesis for the role of sperm-TLR2 in sperm-uterine gland immune interaction is illustrated in **Figure 8**.

The present study mainly focuses on studying the impact of sperm in the uterus giving special emphasis on AI, the technique exclusively used in cattle breeding worldwide. During AI, sperm are directly deposited in the uterus where the amount of SP that accompanies the sperm is reduced since it is significantly diluted using different semen extenders (~1% of SP in frozen semen straws) to maximize the efficiency of a single ejaculate, and routine use of AI reduces maternal exposure to SP (6). Therefore, in the present study, frozen-thawed, washed, and active sperm (via swim-up technique) were used to investigate the impact of sperm-TLR2 on sperm behavior and subsequent penetration into the mucus and uterine glands to trigger the uterine inflammatory response.

The existence of the uterine mucus plays a vital part in supporting sperm passage through the uterus. A viscoelastic mucus exists on the bovine surface endometrium, and sperm have to penetrate this mucus coating to enter the uterine glands (13). Importantly, sperm hyperactivation (i.e., sperm with higher curvilinear velocity, side-to-side head movement, and lower path linearity) (39, 40) facilitates sperm progress through the viscoelastic mucus of the female tract and cumulus oophorus matrix (41).

The involvement of sperm-TLR2 in regulating sperm penetration to oocytes during IVF (28) prompted us to investigate whether the activation of sperm-TLR2 triggers sperm hyperactivation and subsequent mucus penetration. In the present study, the calcium ionophore, a trigger of hyperactivation, increased the percentage of hyperactivated sperm and modulated the mean values of hyperactivated-related parameters (i.e., VCL, ALH, and LIN). The TLR2 activation and blockage increased and decreased the proportion of hyperactivated sperm, respectively. These indicate that the activation of sperm-TLR2 enhances hyperactivated motility, which is associated with the ability to penetrate mucus.

Although the used concentrations of TLR2 agonist or antagonist increased or decreased the sperm hyperactivation, respectively, they did not affect the total motile sperm, mean values of sperm motility parameters, and other functional parameters such as sperm plasma and acrosomal membrane integrity, high mitochondrial membrane potential, and plasma membrane stability throughout the observed time points, which are the prerequisites for their action for fertilization (32, 34, 35). This suggests that sperm-TLR2 partially regulates the motility pattern (i.e., toward hyperactivation) without interfering with other sperm parameters. The detailed mechanism by which sperm-TLR2 regulates the hyperactivation is unclear. However, we have shown previously that the activation of sperm-TLR2 enhances Ca^2+^ influx into sperm (28) which is essential for the induction of hyperactivated sperm movement. Therefore, it is possible that TLR2 activation might modulate the activity of sperm ion channels, such as the CatSper channel, which is responsible for the Ca^2+^ influx and the hyperactivation of sperm (41).

Moreover, the increase in ATP levels is required to induce hyperactivation (41). The present results show that sperm become hyperactivated without changing the mitochondrial membrane potential which is a measure of the ability of the mitochondria to produce ATP. It is possible that the TLR2-activated sperm may have a slight or transient increase in the mitochondrial membrane potential that is sufficient to provide the energy for hyperactivation but not enough to cause significant changes in the mitochondrial membrane. However, more sensitive analysis such as a luciferin-luciferase bioluminescence assay is required to measure the ATP levels to get a deeper understanding of this mechanism. Presumably, these hyperactivated sperm are strong enough to overcome the greater resistance (49, 50) and fluid shear to penetrate through the mucus (51, 52).

In support of the idea above, the proportions of TLR2-activated and -blocked sperm that penetrated the artificial viscoelastic fluid were higher and lower, respectively, compared to non-treated sperm. Importantly, TLR2-activated sperm penetrated the estrous-uterine-mucus in a greater proportion. Finally, in the *ex-vivo* sperm-endometrial explant co-culture model, the fact that the TLR2-activated and -blocked sperm entered the uterine glands with a higher and lower number revealed a clear association between sperm-TLR2 activation and motile activity together with their penetration of mucus and the uterine glands.

More exactly, a lesser portion of non-treated sperm also showed a hyperactivated motile pattern, entered the viscoelastic fluid as well as estrous-uterine-mucus, and penetrated the uterine glands, suggesting that highly active sperm (i.e., hyperactivated sperm) are not exclusive population, but rather a major one to enter the uterine glands. In fact, TLR2-activated sperm showed higher motile activity with hyperactivation after moving into estrous-uterine-mucus, supporting the above interpretation that these activated sperm are the major population detected in the uterine gland. Collectively, our results reveal that TLR2 activation enhances the sperm motile pattern and mucus penetration, thereby contributing to entering into the uterine glands. Since the present *ex-vivo* model has limitations on investigating the features of sperm within the uterine glands, further advanced investigations (such as artificial uterine models) are necessary to clarify which kind of sperm enters the uterine glands in the non-treated sperm group; in particular, it is worth revealing whether the TLR2 is activated in these sperm.

In the uterine explant *ex-vivo* model, the sperm typically trigger the weak inflammatory response with a slight upregulation of *TNFA*, *IL1B*, *IL8*, and *PGES* mRNA expressions (13). Even though a higher percentage of TLR2-activated sperm (by 32% increase vs. TLR2 non-activated sperm) entered the uterine glands, this did not further increase the mRNA expression of the above-investigated cytokines in the present study. This suggests that a certain, but not large, number of sperm acting in the uterine glands is sufficient for triggering the cascade of the maximum physiological inflammatory response toward sperm.

On the other hand, a lesser number of TLR2-blocked sperm (by 44% reduction *vs.* TLR2 non-blocked sperm) in uterine glands weakened the above inflammatory response. Together, the data suggest that a certain number of sperm with the ‘narrow range’ in the uterine gland is sufficient to trigger the physiological uterine inflammatory response. This mechanism of interaction of a small population of sperm with uterine glands might serve to trigger mild and transient inflammatory responses and avoid the induction of massive and generalized inflammation throughout the entire uterine mucosa during sperm transport. Such a weak pro-inflammatory response is essential to switch on the maternal innate immunity, which has a crucial part in eliminating dead and excess sperm in preparing the endometrium without critical damage for implantation (3, 53).

The endogenous ligands to activate the sperm-TLR2 have not been identified in the present study. The endogenous TLR2 ligand such as the extracellular matrix molecule, hyaluronan (23, 54), recognized as a regulator of TLR2 (55), is one of the candidates along with the other possible physiological uterine microbiota-derived ligands (56, 57). Very recently, we reported that hyaluronan was present in the bovine endometrium and modulated the sperm-endometrial epithelial immune interaction through the cluster of differentiation 44 (CD44) and TLR2 (58). Thus, hyaluronan could also assist in activating the sperm-TLR2 *in-vivo*.The TLR1/2 and TLR2/6 are two types of heterodimers that TLR2 forms with either TLR1 or TLR6. These heterodimers have different ligand specificities and signaling pathways, thus having different impacts on the immune response (14). The agonist, Pam3Cys-Ser-(Lys), and antagonist, CU-CPT22, used in the present study are both specific to the TLR1/2 complex. Therefore, it is highly possible that the observed effects are due to the triggering of TLR1/2 heterodimerization of the sperm. In the physiological status, sperm are known to exhibit hyperactivated motility in the oviduct, which is essential for fertilization (41). The present study reveals that hyperactivated sperm also have a significant role in the uterus, at least in cattle, where they penetrate the uterine glands to trigger the uterine immune response. The hyperactivated sperm in the uterus are probably no longer able to migrate toward the oviduct to contribute to fertilization since sperm are exposed to polymorphonuclear neutrophils attack inside the uterine glands (13). Conversely, linear progressive motile sperm in the uterus are known to migrate toward the oviduct to participate in the fertilization process (59) and are also reported to have greater fertilizing capacity (60). These suggest that the inseminated sperm activated by endogenous TLR2 ligands in the uterus probably contribute to triggering the innate immune cascade in the bovine uterus.

In conclusion, our findings provide evidence that, in cattle, sperm-TLR2 activation induces hyperactivation of sperm, successive mucus penetration, and sperm entering into the uterine glands. Thus, it seems that sperm-TLR2 plays a role in the initiation of the whole cascade of sperm-induced uterine inflammatory response. The in-detail binding mechanism of sperm-TLR2 in the direct interaction with the uterine gland epithelium is yet to be intensively investigated.

## Data availability statement

The original contributions presented in the study are included in the article/**Supplementary Material**. Further inquiries can be directed to the corresponding author.

## Ethics statement

The animal study was approved by The Committee on the Ethics of Animal Experiments of the Obihiro University of Agriculture and Veterinary Medicine, Japan (Permit number 27-74). The study was conducted in accordance with the local legislation and institutional requirements.

## Author contributions

IA: Conceptualization, Data curation, Formal Analysis, Investigation, Methodology, Visualization, Writing – original draft, Writing – review & editing. YK: Data curation, Formal Analysis, Investigation, Methodology, Writing – review & editing. TU: Conceptualization, Investigation, Methodology, Resources, Writing – review & editing. CK: Data curation, Formal Analysis, Investigation, Methodology, Software, Writing – review & editing. MSa: Investigation, Methodology, Resources, Supervision, Visualization, Writing – review & editing. MM: Conceptualization, Methodology, Project administration, Supervision, Validation, Writing – original draft, Writing – review & editing. MY: Conceptualization, Data curation, Investigation, Methodology, Supervision, Writing – review & editing. SH: Formal Analysis, Investigation, Methodology, Resources, Visualization, Writing – review & editing. MSh: Conceptualization, Methodology, Project administration, Resources, Supervision, Validation, Writing – review & editing. AM: Conceptualization, Funding acquisition, Methodology, Project administration, Resources, Software, Supervision, Validation, Visualization, Writing – original draft, Writing – review & editing.
