# Supplementary material for: Activation of sperm Toll-like receptor 2 induces hyperactivation to enhance the penetration to mucus and uterine glands: a trigger for the uterine inflammatory cascade in cattle
Source: Front Immunol. 2023 Dec 19;14:1319572. doi: 10.3389/fimmu.2023.1319572 (PMC10766357; doi:10.3389/fimmu.2023.1319572)
Supplement: Supplementary file 1 [file DataSheet_1.docx]

**Supplementary material**

**Activation of sperm Toll-like receptor 2 induces hyperactivation to enhance the penetration to mucus and uterine glands: A trigger for the uterine inflammatory cascade in cattle**

Ihshan Akthar, Yejin Kim, Takashi Umehara, Chihiro Kanno, Motoki Sasaki,

Mohamed Ali Marey, Mohamed Samy Yousef, Shingo Haneda, Masayuki Shimada, Akio Miyamoto*

*Correspondence

Akio Miyamoto

[akiomiya@obihiro.ac.jp](mailto:akiomiya@obihiro.ac.jp)


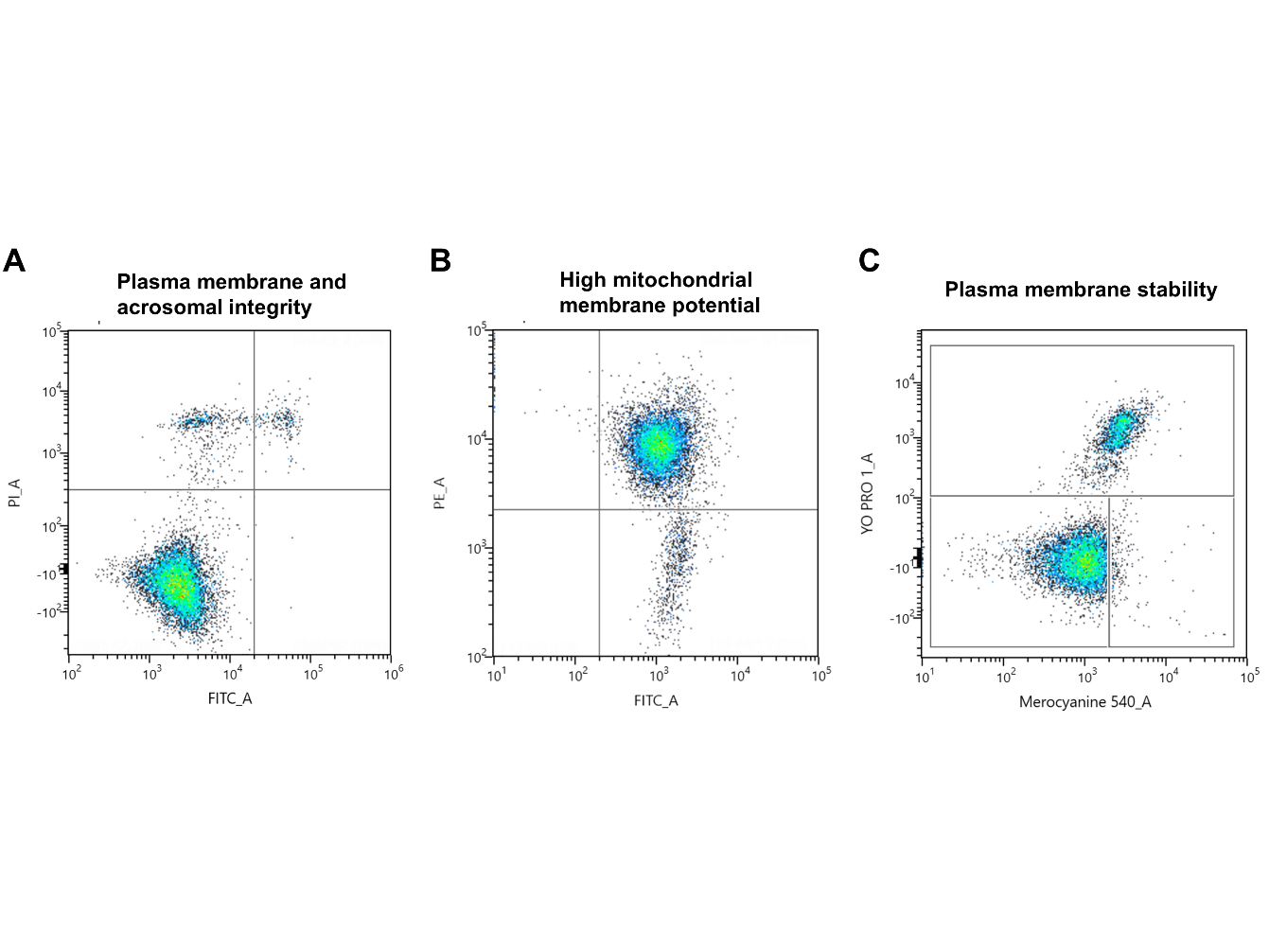
**Supplementary Figure 1.** **A representative flow cytometric dot plot diagram.** **(A)** A representative dot plot diagram of sperm stained with Propidium Iodide (to evaluate membrane integrity) and Fluorescein isothiocyanate-conjugated peanut-agglutinin (to evaluate acrosomal integrity). The panel is divided into four quadrants representing different subpopulations of sperm according to their acrosomal and plasma membrane integrity: the top left quadrant represents the plasma membrane damaged and acrosome intact sperm, the top right quadrant represents the plasma membrane and acrosome damaged sperm, the bottom left quadrant represents the plasma and acrosome membrane intact sperm (PMAI), the bottom right quadrant represents the plasma membrane intact and acrosome damaged sperm. **(B)** A representative diagram of sperm stained with 5,5’,6,6’-tetrachloro-1,1’3,3’-tetraethylbenzimidazolyl-carbocyanine iodide (JC1) fluorescent probe for evaluation of high mitochondrial membrane potential (HMMP). The panel is divided into two sections representing subpopulations of sperm according to their mitochondrial membrane potential: the top section represents the sperm with HMMP, the bottom section represents the sperm with low mitochondrial membrane potential (LMMP). **(C)** A representative diagram of sperm stained with cell viability indicator Yo-Pro 1 and plasma membrane destabilization indicator Merocyanine 540 (M540). The panel is divided into three sections representing subpopulations of sperm according to their viability and plasma membrane stability: the top section represents the sperm that are permeable to Yo-Pro 1 (dead cells), the bottom left section represents the viable sperm with the stable plasma membrane, the bottom right section represents the viable sperm with the instable plasma membrane. Percentages of sperm with stable plasma membrane were calculated out of total viable sperm.

**Supplementary Table 1.** List of the primers used in real-time PCR.

| **Gene** |  | **Sequence of nucleotide (5’🡒3’)** | **Accession no.** | **Fragment size (bp)** |
| --- | --- | --- | --- | --- |
| *B-actin* | Forward | TCACCAACTGGGACGACATG | NM_173979.3 | 51 |
|  | Reverse | CGTTGTAGAAGGTGTGGTGCC |  |  |
| *IL8* | Forward | CCAATGGAAACGAGGTCTGC | NM_173925.2 | 51 |
|  | Reverse | CCTTCTGCACCCACTTTTCCT |  |  |
| *TNFA* | Forward | CAAAAGCATGATCCGGGATG | NM_173966.3 | 51 |
|  | Reverse | TTCTCGGAGAGCACCTCCTC |  |  |
| *IL1B* | Forward | AATCGAAGAAAGGCCCGTCT | NM_174093.1 | 51 |
|  | Reverse | ATATCCTGGCCACCTCGAAA |  |  |
| *PGES* | Forward | AAAATGTACGTGGTGGCCGT | NM_174443.2 | 51 |
|  | Reverse | CTTCTTCCGCAGCCTCACTT |  |  |
| *TLR2* | Forward | CATGGGTCTGGGCTGTCATC | NM_174197.2 | 51 |
|  | Reverse | CCTGGTCAGAGGCTCCTTCC |  |  |

**Supplementary Table 2.** Flow cytometry analysis to detect the effects of TLR1/2 agonist (TLR2 activation) on the sperm plasma membrane and acrosomal integrity (PMAI), high mitochondrial membrane potential (HMMP), and plasma membrane stability of frozen-thawed active (swim-up) sperm. Results are presented as means ± SEM. TLR2 ago.^(-)^ versus TLR2 ago.^(+)^ were compared at the same points.

| Sperm parameters | Treatment | 0 min | 30 min | 2 h |
| --- | --- | --- | --- | --- |
| PMAI % | TLR2 ago.^(-)^ | 83.74 ± 2.60 | 82.05 ± 2.92 | 74.25 ± 3.27 |
|  | TLR2 ago.^(+)^ | 82.19 ± 3.01 | 80.51 ± 3.84 | 72.46 ± 3.83 |
| HMMP % | TLR2 ago.^(-)^ | 87.26 ± 1.91 | 86.52 ± 2.10 | 87.87 ± 1.13 |
|  | TLR2 ago.^(+)^ | 87.00 ± 1.20 | 88.01 ± 1.53 | 89.95 ± 1.72 |
| Viable sperm %  (Yo-Pro 1 negative) | TLR2 ago.^(-)^ | 74.07 ± 2.29 | 71.52 ± 2.08 | 68.85 ± 4.25 |
|  | TLR2 ago.^(+)^ | 73.38 ± 1.83 | 72.13 ± 2.36 | 68.95 ± 3.01 |
| Stable plasma membrane/viable sperm % | TLR2 ago.^(-)^ | 88.18 ± 1.85 | 87.45 ± 1.75 | 80.07 ± 2.62 |
|  | TLR2 ago.^(+)^ | 87.03 ± 1.84 | 86.67 ± 1.14 | 81.28 ± 1.67 |

**Supplementary Table 3.** Flow cytometry analysis to detect the effects of TLR1/2 antagonist (TLR2 blockage) on the sperm plasma membrane and acrosomal integrity (PMAI), high mitochondrial membrane potential (HMMP), and plasma membrane stability of frozen-thawed active (swim-up) sperm. Results are presented as means ± SEM. TLR2 ant.^(-)^ versus TLR2 ant.^(+)^ were compared at the same points.

| Sperm parameters | Treatment | 0 min | 30 min | 2 h |
| --- | --- | --- | --- | --- |
| PMAI % | TLR2 ant.^(-)^ | 88.68 ± 1.03 | 85.69 ± 1.32 | 78.78 ± 2.21 |
|  | TLR2 ant.^(+)^ | 89.90 ± 0.63 | 87.10 ± 1.20 | 77.31 ± 2.52 |
| HMMP % | TLR2 ant.^(-)^ | 88.56 ± 1.97 | 88.80 ± 1.00 | 87.67 ± 2.19 |
|  | TLR2 ant.^(+)^ | 87.63 ± 1.97 | 86.19 ± 1.23 | 83.76 ± 3.93 |
| Viable sperm %  (Yo-Pro 1 negative) | TLR2 ant.^(-)^ | 79.61 ± 0.76 | 77.49 ± 1.27 | 71.91 ± 3.74 |
|  | TLR2 ant.^(+)^ | 81.11 ± 0.78 | 78.47 ± 1.42 | 73.96 ± 1.61 |
| Stable plasma membrane/viable sperm % | TLR2 ant.^(-)^ | 94.80 ± 0.71 | 95.92 ± 0.36 | 85.57 ± 4.73 |
|  | TLR2 ant.^(+)^ | 96.54 ± 0.34 | 96.90 ± 0.29 | 90.53 ± 3.10 |

**Supplementary Table 4.** CASA analysis to detect the effects of TLR1/2 agonist (TLR2 activation) on motion parameters of frozen-thawed active (swim-up) sperm. Straight-line velocity (VSL), curvilinear velocity (VCL), average path velocity (VAP), linearity (LIN), straightness (STR) beat cross frequency (BCF), and amplitude of lateral head (ALH). Results are presented as means ± SEM. TLR2 ago.^(-)^ versus TLR2 ago.^(+)^ were compared at the same points.

| Sperm Parameters | Treatment | 0 h | 0.5 h | 2 h |
| --- | --- | --- | --- | --- |
| Total motile sperm (%) | TLR2 ago.^(-)^ | 92.47 ± 2.58 | 94.63 ± 1.53 | 90.37 ± 2.56 |
|  | TLR2 ago.^(+)^ | 94.92 ± 1.23 | 93.19 ± 0.55 | 92.84 ± 1.56 |
| Progressive motile sperm (%) | TLR2 ago.^(-)^ | 74.85 ± 1.49 | 75.23 ± 2.28 | 72.96 ± 2.39 |
|  | TLR2 ago.^(+)^ | 73.45 ± 1.30 | 73.27 ± 1.36 | 71.18 ± 1.51 |
| VSL (µm/s) | TLR2 ago.^(-)^ | 127.48 ± 7.04 | 123.11 ± 7.23 | 111.40 ± 6.38 |
|  | TLR2 ago.^(+)^ | 123.73 ± 6.89 | 121.30 ± 6.49 | 111.36 ± 6.75 |
| VCL (µm/s) | TLR2 ago.^(-)^ | 284.69 ± 9.38 | 280.92 ± 12.84 | 239.51 ± 10.56 |
|  | TLR2 ago.^(+)^ | 288.34 ± 9.38 | 276.87 ± 10.24 | 248.62 ± 13.12 |
| VAP (µm/s) | TLR2 ago.^(-)^ | 140.47 ± 7.38 | 136.89 ± 8.25 | 122.08 ± 7.92 |
|  | TLR2 ago.^(+)^ | 140.10 ± 7.41 | 134.20 ± 7.44 | 124.92 ± 8.69 |
| LIN (%) | TLR2 ago.^(-)^ | 43.00 ± 1.33 | 41.51 ± 0.71 | 43.90 ± 0.65 |
|  | TLR2 ago.^(+)^ | 41.11 ± 1.20 | 40.99 ± 0.74 | 42.55 ± 0.63 |
| STR (%) | TLR2 ago.^(-)^ | 87.15 ± 1.30 | 86.10 ± 0.85 | 87.25 ± 0.70 |
|  | TLR2 ago.^(+)^ | 84.93 ± 0.91 | 85.48 ± 0.88 | 85.23 ± 0.65 |
| ALH (µm) | TLR2 ago.^(-)^ | 4.13 ± 0.09 | 4.33 ± 0.09 | 3.86 ± 0.12 |
|  | TLR2 ago.^(+)^ | 4.30 ± 0.11 | 4.33 ± 0.05 | 3.96 ± 0.16 |
| BCF (Hz) | TLR2 ago.^(-)^ | 15.29 ± 1.65 | 14.33 ± 1.28 | 13.71 ± 1.29 |
|  | TLR2 ago.^(+)^ | 15.14 ± 1.60 | 13.66 ± 1.35 | 13.78 ± 1.35 |

**Supplementary Table 5.** CASA analysis to detect the effects of TLR1/2 antagonist (TLR2 blockage) on motion parameters of frozen-thawed active (swim-up) sperm. Straight-line velocity (VSL), curvilinear velocity (VCL), average path velocity (VAP), linearity (LIN), straightness (STR) beat cross frequency (BCF), and amplitude of lateral head (ALH). Results are presented as means ± SEM. TLR2 ant.^(-)^ versus TLR2 ant.^(+)^ were compared at the same points.

| Sperm Parameters | Treatment | 0 h | 0.5 h | 2 h |
| --- | --- | --- | --- | --- |
| Total motile sperm (%) | TLR2 ant.^(-)^ | 97.91 ± 0.90 | 97.28 ± 1.05 | 93.57 ± 1.14 |
|  | TLR2 ant.^(+)^ | 97.07 ± 0.97 | 95.17 ± 0.87 | 91.37 ± 1.68 |
| Progressive motile sperm (%) | TLR2 ant.^(-)^ | 78.22 ± 3.46 | 71.99 ± 1.62 | 72.70 ± 2.31 |
|  | TLR2 ant.^(+)^ | 75.30 ± 1.50 | 76.80 ± 2.14 | 69.96 ± 1.98 |
| VSL (µm/s) | TLR2 ant.^(-)^ | 134.47 ± 2.80 | 133.86 ± 5.32 | 118.45 ± 6.08 |
|  | TLR2 ant.^(+)^ | 131.60 ± 5.23 | 130.89 ± 3.24 | 108.03 ± 6.51 |
| VCL (µm/s) | TLR2 ant.^(-)^ | 298.64 ± 7.10 | 303.44 ± 7.51 | 250.66 ± 11.38 |
|  | TLR2 ant.^(+)^ | 292.29 ± 8.09 | 289.57 ± 6.06 | 231.17 ± 11.74 |
| VAP (µm/s) | TLR2 ant.^(-)^ | 149.25 ± 4.02 | 150.81 ± 5.18 | 128.16 ± 7.10 |
|  | TLR2 ant.^(+)^ | 146.01 ± 5.24 | 144.02 ± 2.98 | 119.19 ± 7.29 |
| LIN (%) | TLR2 ant.^(-)^ | 45.28 ± 0.93 | 43.70 ± 0.87 | 45.25 ± 0.72 |
|  | TLR2 ant.^(+)^ | 44.90 ± 1.24 | 44.07 ± 0.45 | 43.51 ± 1.13 |
| STR (%) | TLR2 ant.^(-)^ | 89.02 ± 0.93 | 88.10 ± 1.15 | 89.47 ± 0.76 |
|  | TLR2 ant.^(+)^ | 88.55 ± 1.17 | 88.55 ± 0.75 | 85.71 ± 1.43 |
| ALH (µm) | TLR2 ant.^(-)^ | 4.18 ± 0.17 | 4.51 ± 0.09 | 3.96 ± 0.17 |
|  | TLR2 ant.^(+)^ | 4.14 ± 0.12 | 4.32 ± 0.12 | 3.73 ± 0.18 |
| BCF (Hz) | TLR2 ant.^(-)^ | 17.48 ± 1.86 | 16.02 ± 1.41 | 13.77 ± 1.21 |
|  | TLR2 ant.^(+)^ | 17.33 ± 1.97 | 15.64 ± 1.16 | 13.97 ± 1.13 |

**Supplementary Table 6.** The sperm motion parameters of control and A23187-treated sperm *via* CASA analysis. Straight-line velocity (VSL), curvilinear velocity (VCL), average path velocity (VAP), linearity (LIN), straightness (STR) beat cross frequency (BCF), and amplitude of lateral head (ALH). Results are presented as means ± SEM of three independent experiments. *p<0.05 denotes the significant difference between the control and A23187-treated group.

| Sperm Parameters | Control | A23187 |
| --- | --- | --- |
| Total motile sperm (%) | 82.66 ± 2.11 | 86.93 ± 1.63 |
| Progressive motile sperm (%) | 57.99 ± 3.42 | 65.21 ± 1.67 |
| VSL (µm/s) | 102.10 ± 5.52 | 102.51 ± 1.40 |
| VCL (µm/s) | 233.23 ± 2.06 | 260.17 ± 5.47* |
| VAP (µm/s) | 111.73 ± 5.49 | 117.01 ± 1.90 |
| LIN (%) | 40.68 ± 0.41 | 38.03 ± 0.52* |
| STR (%) | 85.03 ± 1.02 | 84.35 ± 0.35 |
| ALH (µm) | 3.89 ± 0.04 | 4.16 ± 0.05* |
| BCF (Hz) | 11.59 ± 0.36 | 11.37 ± 0.15 |
